# Dentistry in the Twenties

**Published:** 1898-05

**Authors:** D. D. Atkinson


ARTICLE VII.

### DENTISTRY IN THE TWENTIES.

D. D. ATKINSON, D. D. S.

**Extracts from the Works of Samuel S. Filch.**

Thinking it would be interesting to "The Weekly" readers, I have spent a brief season with the teachings of the above author, who is accredited as being one of the lights of his day, and who no doubt gives us a faithful portrayal of our science at that time. Few, if any, shall be my comments on his teachings, but will leave to those who read these extracts to say if the people should appreciate what an intelligent application of scientific principles in our profession has done for them.

### TREATMENT OF COMPLICATED CASES.

"Attempts to destroy the inflamed nerves of teeth, especially the molar and bicuspid, are not only very painful, but most usually ineffectual. If we succeed in destroying the nerve, the tooth is often rendered almost useless.

I here subjoin a case of an unsuccessful attempt to destroy the nerves of a diseased tooth, which came under the observation of my friend, Mr. Eleasor Parmley, of New York. 'In a front tooth the nerve is most commonly destroyed by a single operation, because the fang is single and has the advantage of being more perpendicular than in a tooth with divaricating fangs. But it is an erroneous idea that a diseased tooth, if it has more than a single fang, may be rendered useful and free from pain by destroying its nerves. The practice has only served to expose the emptiness of the theory, since most of those who have undergone the operation, which can be termed little less than martyrdom, have barely found that they have been made to forget the usual pain of toothache in the unutterable agony of the operation. But this is not all the objection; for where the operator is so fortunate as partially to destroy the nerves of double teeth, as even this is very rarely the case, the membranes are apt to become diseased by inflammatory action, and the tooth requires to be extracted in a very short time afterwards. It cannot therefore be too strongly urged, that where a double tooth is painful and has become so much decayed as not to be capable of being saved by the operation of stopping, it should, in order to prevent all unpleasant consequences, be extracted immediately. In evidence of the fallacy of the attempts of destroying the nerves of the back teeth, I shall adduce a single instance, which came under my own observation.

"A gentleman possessing highly organized teeth, having twice suffered very serious lacerations of the bone from extraction, and having even been threatened with lockjaw, submitted to having the fangs of the first lower molar, which had long been a source of torture, drilled, with the hope of thus eradicating its nerves. The operation, after excruciating agonies, proved within a few hours to have been useless; the cavity of the tooth was then filled with a compound metallic stopping; but the pain returned with such violence that it was necessary to remove it. The patient continued during many months to make every application and adopt every measure which the most experienced medical practitioners could suggest, but in vain. His protracted suffering brought on a low fever, accompanied by frequent delirium. Efforts were again and again made at extraction; but at the first touch of an instrument the patient was always seized with convulsions and the operation could not be effected. Having thus lingered on for six months, the tooth was fortunately extracted during a period of insensibility, the result of intense suffering; but although the expected local relief was thus obtained, several months elapsed before he regained his former health and vigor. The tooth was examined after extraction, when it appeared that very trifling portions of the nerve had been destroyed, that one fang contained a large and vigorous nerve, sending off five branches at its point; the other fang, a large nerve equally unaltered, sending off six branches around its point.'

"All these modes of destroying the nerve occasion great pain to the patient in most cases, and what is still more objectionable, by destroying the nerve, the rest of the vitality of the crown and body of the tooth is soon lost, and, at any rate, those parts change their côlor and often incline to produce a diseased state of the gums and remaining teeth; although bad consequences may arise to the patient from this course, still in some cases, from the desire of the patient or our own inclination, we may do it in preference to extracting the tooth. After destroying the nerve we should immediately plug the tooth and leave it to nature."

### OF SUBSTANCES PROPER FOR FILLING THE CAVITIES OF THE TEETH.

"It would seem superfluous to speak particularly upon this subject." (If this subject was thread-bare in that day, what may be said of it to-day; or considering the bent and genius of the human mind, its achievements and possibilities, may it not be said that, comparatively speaking, this same subject is as embryonic now as then? "A.")

"One would suppose that the common sense and discrimination of men, and practitioners in particular, would determine this point after the years of experience that have elapsed since plugging the teeth has been practiced. But such is the ignorance or cupidity of some dentists at this period, that there are not wanting men who will assert to their patients that lead or tin is better than gold, and thereby impose in the grossest manner upon their patients and the public in general. Of this, as an illustration, I will mention the following case: Two young gentlemen who were preparing for the Christian ministry called on me in May, 1827, to consult me respecting their teeth. I found in both that their teeth were in a very bad condition. The front incisors of both were in a state of decay. I advised a course of dental operations, which would place their teeth and gums in a state of health, and that the carious front teeth should be plugged with gold; and more especially so, because they were intending to become public speakers, in whom the health and beauty of the teeth are indispensable to a cleanly appearance of their mouths, and perfect enunciation of language.

"They both went away, saying if they concluded to have their teeth operated upon they would call soon. One of them called in two or three days, and by a judicious course of operations on the principles before detailed and which will be hereafter, I rendered his teeth perfectly healthy. The upper incisors, two or three, I plugged with gold in a very perfect manner, so that I saw him in the next November and his teeth were all in fine order and health.

"While plugging his teeth he told me that his friend who came with him at first to ask my advice, had called upon another dentist who persuaded him that tin-foil was far better than gold for plugging the teeth, that it was retained longer and was in all respects quite preferable to gold; and so little was the young gentleman acquainted with the subject, that he suffered the dentist to plug his teeth with tin foil and two or three of his incisors, one of which he said was so far decayed that he thought best to leave it to ultimate destruction. Now any person can see the result of this case. A gradual exidation to a certain extent of the tin immediately commences which gives the substance of the tooth opposite the plug a dark and repulsive appearance, and what is far worse beyond comparison, that by the gradual oxidation of the tin the caries of the tooth is suffered to go on, favored by the oxidizing metal; and ultimate destruction of the tooth is the inevitable consequence; while those as in the first case plugged with gold will remain in all probability external caries excepted, during the life of the patient precisely as when first introduced. It is but a few weeks since I saw the front incisor, shown me by a young lady, which had been plugged about two years, and with lead, in which the lead was almost completely oxidated and the caries had proceeded so far as to entirely destroy the vitality, and almost the substance of the tooth, so much so that I could pass a probe directly through the front surface of the tooth which was black and a mere shell She told me that when the tooth was plugged it was but little decayed.

"Caries of this kind are seen every day, and yet some dentists have the hardihood to assert that tin and lead are as proper for plugging the teeth as gold, although be it said, to the credit of the profession, that very few respectable dentists are guilty of this abominable ignorance or dishonesty. Lead may be applied, as we have before mentioned, to cover the nerve of the tooth, and the filling completed with gold. Tin foil, if pure, may be used in some cases for plugging the grinding-teeth if the patients are not able to pay for gold, or if the gold cannot be obtained."

### METHODS OF RETAINING ARTIFICAL TEETH IN THE MOUTH.

"Ligatures.—In this case we should have to continue our block of teeth to the last molar tooth or not be able to use a ligature, and should we do so, a ligature would not confine such a large block of teeth as firmly as they ought to be. In the next place, if made of silk, etc., they are extremely apt to contract an unpleasant taste and smell, become dirty, and the patient is obliged to change them very often. If made of gold or silver wire, then the patient will be troubled to unite them, so as to take out the teeth and clean them as he will desire to do occasionally, and he will be troubled to tie them in again. In the third place, they never give that firmness in the new teeth which we desire and the teeth are constantly moved by the tongue. The fourth and last objection I would mention to ligatures and one which, if we could pass by all others, would forever in most cases forbid their use altogether, is the injurious effects which ligatures suspending the teeth have upon those living teeth to which they are tied. Their first bad effect is to puc those teeth out to which they are tied, which they generally do sooner or later, or else they cut off the tooth, as I have seen done in repeated instances."

### PHYSIOLOGICAL OBSERVATIONS UPON STUMPS OF TEETH, ETC.—MODE OF PERFORMING THE OPERATION, INSTRUMENTS, ETC.

"The instruments required are a pair of sharp-cutting forceps, like extracting forceps, only attenuated to sharp edges, and well tempered. These should be placed upon the tooth to be excised, and carried close to the gum, and even raise the gum a little, or depress it, as the case may be, when, with a steady, deliberate pressure upon the handles of the forceps, the tooth is instantly cut off; then, with a pointed instrument, we may remove and destroy the nerve. Having done this, if we wish to engraft a new tooth, we may drill up the stump as much as will be necessary for the reception of a pivot; but we must not at this time insert the tooth; instead of which we put a small lead pivot in the fang so firmly that it will not drop out; we may then dismiss our patient for at least one week, with directions to avoid taking cold or heating himself much during that time. At the expiration of seven or eight days, if little or no inflammation has appeared, we may take out the lead pivot and engraft the new tooth. In cases where the tooth is so far decayed or crumbled away as not to require much cutting away, and where the lining membrane and nerve are dead, if we wish to engraft a tooth upon the stump we may drill it out as much as we wish to, and then insert our leaden pivot, as before directed; for if we neglect this precaution, inflammation, in a great many instances, will take place, so as often to compel us to remove the engrafted tooth, or at any rate it will greatly weaken the stump and occasion much suffering to the patient. It should be our earnest endeavor to save pain to our patients as much as possible which will be done to a great degree by pursuing the practice I have here detailed. The many cases of most violent inflammation and extreme suffering occasioned by this mode of inserting teeth upon stumps without being prepared (so much so in many cases as to almost destroy the constitution and health of the patients) has often thrown this part of dental surgery into great disrepute, and has been a standing opprobrium to the profession. These wretched effects in every case when I have followed this plan, have been completely prevented, and the whole course of operation has not produced as much pain as the extraction of the tooth or stump would have done. Other modes of preparing stumps have been practiced, but I prefer this to any other. If the patients do not wish a new tooth to be engrafted, then we need not drill the fang, but fill the internal cavity with a piece of soft, pure lead wire, so firmly introduced as that it will not come out unless done so by art. Little or no pain will ensue, and the patient's mouth will retain its form. I believe that this plan of treating the front teeth and of preparing stumps of teeth for insertion of artificial teeth is founded upon the most correct surgical principles, and if generally adopted will disarm this part of dental surgery of nearly all its terrors.

"We should, before inserting a new tooth upon a stump, cut away the end of the stump so that the end of the new tooth shall pass completely within the gum and firmly against the end of the stump. When so done this is the best mode of inserting artificial teeth with which we are at present acquainted."

### ODONTALGIC PILLS.

"In applying medicines to the exposed nerves of the teeth, either to relieve toothache or prepare the nerves of the teeth for receiving the presence of metallic stoppings, it is often convenient and indispensable to form our medicines into pills, so as to fill the cavity of a tooth in this way, from which fluid medicines would immediately pass off. I give a few forms of these. In their exhibition, the pills are to be adapted to the size of the cavity in the decaying tooth, and after introduced it should be covered with a piece of wax, so as to prevent the pill from being dissolved into the liquor of the mouth :

℞

| | |
|---|---|
| Pulvis gallæ, . . | ʒ ii. |
| Opium, . . . | ʒ ss. |
| Pulvis camphoræ, . . | ʒ jss. |
| Tinc. daturæ stramonii, . | q. s. |

Reduce the substance to pills.

—*American Dental Weekly.*

